# Simulation and Analysis of Tethering Behavior of Neutrophils with Pseudopods

**DOI:** 10.1371/journal.pone.0128378

**Published:** 2015-06-19

**Authors:** Anne D. Rocheleau, Ronen Sumagin, Ingrid H. Sarelius, Michael R. King

**Affiliations:** 1 Department of Biomedical Engineering, Cornell University, Ithaca, New York, United States of America; 2 Department of Pathology, Feinberg School of Medicine, Northwestern University, Chicago, Illinois, United States of America; 3 Department of Pharmacology and Physiology, University of Rochester Medical Center, Rochester, New York, United States of America; The University of Akron, UNITED STATES

## Abstract

P-selectin and P-selectin glycoprotein ligand-1 (PSGL-1) play important roles in mediating the inflammatory cascade. Selectin kinetics, together with neutrophil hydrodynamics, regulate the fundamental adhesion cascade of cell tethering and rolling on the endothelium. The current study uses the Multiscale Adhesive Dynamics computational model to simulate, for the first time, the tethering and rolling behavior of pseudopod-containing neutrophils as mediated by P-selectin/PSGL-1 bonds. This paper looks at the effect of including P-selectin/PSGL-1 adhesion kinetics. The parameters examined included the shear rate, adhesion on-rate, initial neutrophil position, and receptor number sensitivity. The outcomes analyzed included types of adhesive behavior observed, tether rolling distance and time, number of bonds formed during an adhesive event, contact area, and contact time. In contrast to the hydrodynamic model, P-selectin/PSGL-1 binding slows the neutrophil’s translation in the direction of flow and causes the neutrophil to swing around perpendicular to flow. Several behaviors were observed during the simulations, including tethering without firm adhesion, tethering with downstream firm adhesion, and firm adhesion upon first contact with the endothelium. These behaviors were qualitatively consistent with *in vivo* data of murine neutrophils with pseudopods. In the simulations, increasing shear rate, receptor count, and bond formation rate increased the incidence of firm adhesion upon first contact with the endothelium. Tethering was conserved across a range of physiological shear rates and was resistant to fluctuations in the number of surface PSGL-1 molecules. In simulations where bonding occurred, interaction with the side of the pseudopod, rather than the tip, afforded more surface area and greater contact time with the endothelial wall.

## Introduction

During inflammation, tissue mediators release cytokines that attract free-flowing neutrophils in the bloodstream. This causes neutrophils to tether and roll on the endothelium lining blood vessels before extravasating into the inflamed site [1]. Selectins mediate the capture of free-flowing neutrophils to the inflamed blood vessels. The selectins are a family of cell-surface glycoproteins that includes E-, L- and P-selectin. Selectins show a significant degree of sequence homology among themselves (except in the transmembrane and cytoplasmic domains) and between species. Selectins have an N-terminal lectin domain, an epidermal growth factor (EGF) domain, two (L-selectin), six (E-selectin) or nine (P-selectin) consensus repeats, a transmembrane domain, and a cytoplasmic domain [2]. All of the selectins bind with P-selectin glycoprotein ligand-1 (PSGL-1) and have additional receptors [3]. PSGL-1, concentrated on the tips of surface protrusions called microvilli [4], accounts for 90% of P-selectin binding [2]. The structure of PSGL-1 includes an N-terminal tyrosine sulfate, a long glycoprotein backbone, a transmembrane protein, and a short cytoplasmic tail [2]. Cell adhesion is increased when the thickness of the glycocalyx lining the endothelium is lessened [5].

**Fig 1:**
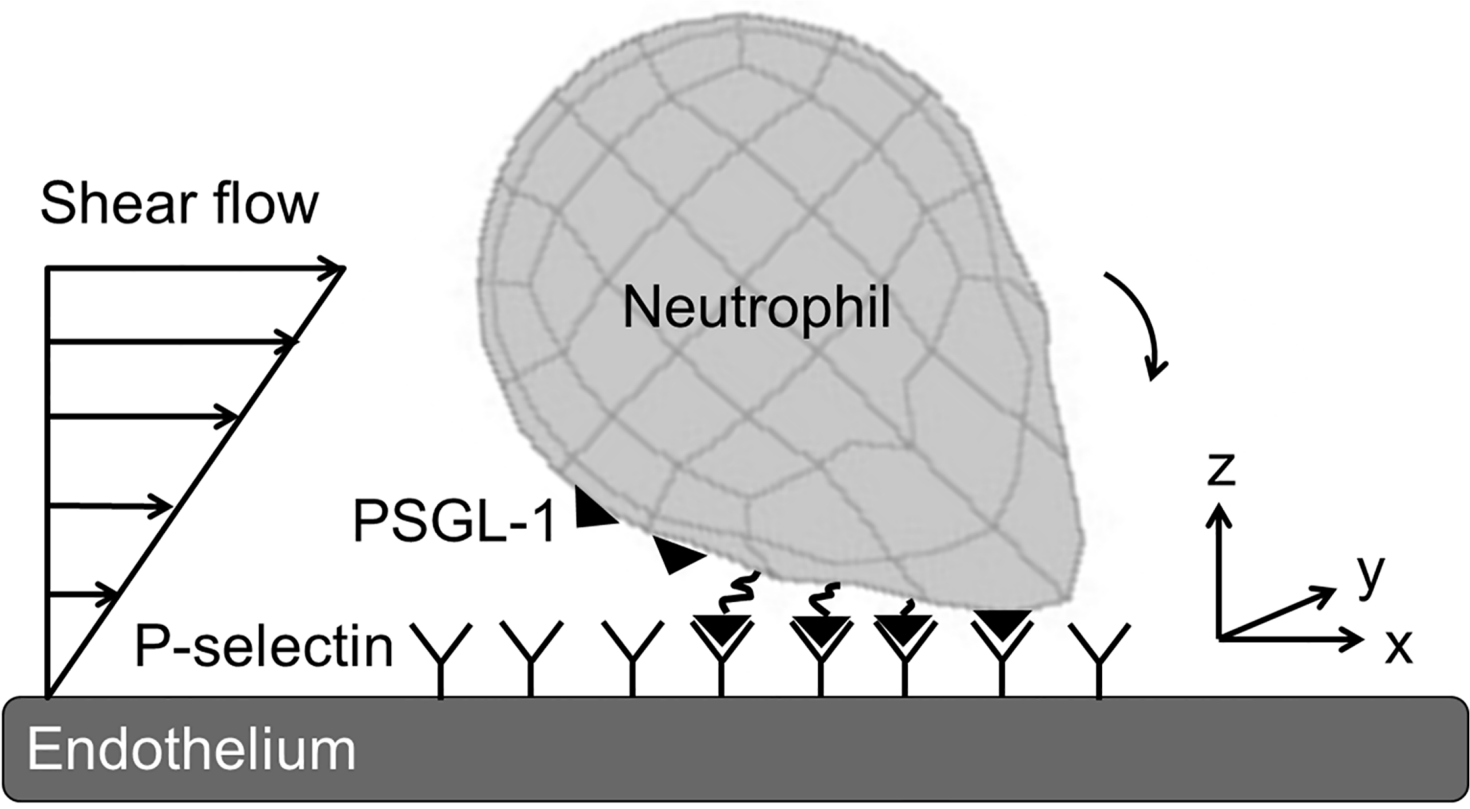
Diagram of neutrophil with pseudopod under shear flow interacting with the endothelium. The neutrophil is represented by a spherical body and a Gaussian-shaped pseudopodial extension, and the endothelium is represented as a semi-infinite wall. PSGL-1 is constitutively expressed on the neutrophil’s surface and binds to P-selectin on the inflamed endothelium. Neither the PSGL-1 nor the P-selectin affect hydrodynamics.

During inflammation, neutrophils extrude organelle-less pseudopods (also called lamellipods) that are essential in crawling on and eventually extravasating through the endothelium. Pseudopod formation has been implicated in a number of diseases, including stroke, coronary disease, and peripheral vascular disease [6]. This process can happen when neutrophils are in the bloodflow or bound to the endothelium [7]. Once extended, pseudopods are relatively stiff formations due to the actin filaments that provide structure [8,9]. Since only 10% of activated, migrating neutrophils transmigrate into the extravascular space and the vast majority detach from the wall and rejoin the blood flow, there is an unstudied population of circulating neutrophils with stable pseudopods. Indeed, pseudopods were even exhibited by four percent of leukocytes in whole blood collected from healthy humans [10]. Typically, neutrophils retract pseudopods when stimulated with shear stress [11]; however, centrifuged leukocytes and those treated with glucocorticoid, an anti-inflammatory cytokine, have been shown to retain their pseudopods under shear stress [12,13]. Here, we analyze the adhesion kinetics of this unstudied population of circulating neutrophils with stable pseudopods. This paper incorporates P-selectin/PSGL-1 binding kinetics into a hydrodynamic model. When a neutrophil has formed bonds with the endothelium, the shear flow exerts a hydrodynamic force on the rear of the cell (Fig 1). The stressed bonds at the back of the cell then break, causing the cell to move forward and form new bonds on the underside of the cell. The balance between shear flow, new bond formation, and bond breakage causes the neutrophil to tether and roll on the endothelium [14]. A more detailed understanding of the impact of neutrophil shape on transport behavior could yield opportunities for potential therapies that modulate the inflammatory cascade.

## Materials and Methods

To better understand the complex process of cell tethering and rolling under flow, the three-dimensional computational model Multiparticle Adhesive Dynamics (MAD) was used to simulate both the hydrodynamic motion and selectin kinetic components [15]. MAD has been utilized previously for spherical rolling neutrophils and has been adapted for multiple spherical particles [16–20] and for platelet behavior, represented as an oblate spheroid [21–24]. The hydrodynamic methodology is described in detail in prior publications. For this study, a Gaussian-shaped extension was projected out of one side of the sphere to represent a neutrophil body with pseudopod with two potential points of contact between the neutrophil and the wall. The pseudopod length was set to be 1.9 times the spherical body radius. The neutrophil with pseudopod had an equal volume to the spherical neutrophil of radius 4 μm used in recent MAD neutrophil studies [25,26]. The endothelial wall was modeled as a plane and the shear rate was 1000 s^-1^ unless otherwise specified. Prior MAD studies have looked into several selectin/receptor pairs, including P-selectin/PSGL-1 kinetics, so this was used in this study for straightforward comparison.

### Multiparticle adhesive dynamics

Bonds were allowed to form between P-selectin on the wall and PSGL-1 on the neutrophil surface. There was a total of 25,100 PSGL-1 receptors on the neutrophil’s surface for a density of 125 molecules/μm^2^; this value was based on the physiological range given by King and Hammer [15]. The number of receptors allotted to each of the 384 elements in the QUAD9 mesh was proportional to its area. Steric layers were added to the cell and wall (0.175 μm and 0.35 μm, respectively) to represent the roughness of the cell membrane and the glycocalyx molecules on the cell surface [15]. The maximum number of bonds allowed to form between the neutrophil and endothelium was set to a large number; 200 was sufficient to avoid limitations to spontaneous bond formation. In the MAD model, the Monte-Carlo method was employed in which two probability equations determine the likelihood at each time step of bond formation (*P*_*f*_) and breakage (*P*_*r*_), respectively:

$$P_{f}=1-e^{-k_{f}\Delta t}$$ (1)

$$P_{r}=1-e^{-k_{r}\Delta t}$$ (2)

where *k*_*f*_ and *k*_*r*_ are the bond formation and dissociation rates, respectively, and ∆*t* is the simulation time step.

#### Bond formation

The bond formation rate depends on the bond length and the slip velocity between two bonding molecules and comes from Bell’s expression of the equilibrium constant for cell-cell bond bridging [27]:

$$k_{f}=k_{f,2-D}^{0}v_{s}\exp\left(\sigma \left|x_{b}-l_{b}\right|\frac{\gamma -0.5\left|x_{b}-l_{b}\right|}{k_{B}T}\right)$$ (3)

where $k_{f,2-D}^{0}$ is the unstressed formation rate constant, *v*_*s*_ is the slip velocity determined by the rotational and translational velocities as well as the location of the PSGL-1 receptor on the neutrophil surface. The bonds were modeled as linearly-elastic springs, with *σ* representing the bond spring constant. The bond length |*x*_*b*_ − *l*_*b*_| results from the absolute value of the difference between the centroid height and the distance from the tip of the PSGL-1 receptor on the neutrophil surface to the steric layer on the endothelium. *k*_*B*_ *T* is the product of the Boltzmann constant and temperature. The values for, *σ*, *l*_*b*_, and *γ* were taken from experimental values used in spherical MAD neutrophil simulations [15,25] and are found in Table 1. $k_{f,2-D}^{0}$ has not been determined experimentally, so it was probed as a parameter for study and a value of 10 s^-1^ was chosen for use for all other simulations.

**Table 1:** Values of bond formation kinetic parameters.

Symbol	Parameter	Value	Reference
*l*_*b*_	Equilibrium P-selectin/PSGL-1 bond length	80 nm	[25]
*σ*	Bond spring constant	250,000 fN/ μm	[25]
$k_{f,2-D}^{0}$	Unstressed rate of bond formation^a^	Varies; default is 10 s^-1^	
*γ*	Reactive compliance	3.9×10^−5^ μm	[28]

The bond formation kinetic parameters used in the MAD model are given here.

^a^The unstressed rate of bond formation is not experimentally determined, so it is explored as an additional variable.

#### Bond dissociation

The bond dissociation rate follows the two-pathway model proposed by Evans et al. [29]. In the model, there are two structure-dependent possible failure pathways [14]. The first is the “catch” pathway, or native conformation (NG), which dominates at low forces and has a fast dissociation rate. In this regime, bonds resist breakage and become stronger; thus, the dissociation rate is constant. The second pathway is the “slip” regime, or intermediate conformation (IG), which dominates in high shear and has a slower bond dissociation rate. The parameter values are found in Table 2. The slip bond dissociation kinetics are represented by the Bell model [30]:

$$k_{r}=k_{r}^{0}\;\exp\left(\frac{\gamma \sigma \left|x_{b}-l_{b}\right|}{k_{B}T}\right)$$ (4)

Both pathways are included in the full dissociation rate equation:

$$k_{r}=\frac{1}{1+\Phi }k_{N}(f)+\frac{\Phi }{1+\Phi }k_{I}(f),\Phi =IG/NG$$ (5)

where *k*_*N*_ is the catch regime dissociation rate, and *k*_*I*_ is the slip pathway dissociation rate. Φ is force-dependent and represents the population ratio of binding molecules between the two pathways:

$$\Phi (f)=\frac{k_{NG\to IG}^{0}}{k_{IG\to NG}^{0}}\exp\left(\frac{\gamma 'f}{k_{B}T}\right)$$ (6)

where $\frac{k_{NG\to IG}^{0}}{k_{IG\to NG}^{0}}$ is the unstressed equilibrium constant for NG-IG states, and *γ*' is the force compliance ratio for the two pathways. The native conformation is independent of force:

$$k_{N}(f)=k_{N,off}^{0}$$ (7)

where $k_{N,off}^{0}$ is the intrinsic dissociation constant. The intermediate or slip pathway follows the quasi-first-order equation:

$$k_{I}(f)=k_{I,off}^{0}\exp\left(\frac{y_{I}^{}f}{k_{B}T}\right)$$ (8)

where $k_{I,off}^{0}$ is the intrinsic dissociation constant and *y*_*I*_ is the force compliance of dissociation for the IG state.

**Table 2:** Values of bond dissociation kinetic parameters.

Symbol	Parameter	Value	Reference
$k_{N,off}^{0}$	Unstressed bond dissociation rate for catch pathway	9 s^-1^	[25]
$k_{I,off}^{0}$	Unstressed bond dissociation rate for slip pathway	0.33 s^-1^	[25]
*y*_*I*_	Force dependence of dissociation for slip pathway	2.4×10^−4^ μm	[28]
$\frac{k_{NG\to IG}^{0}}{k_{IG\to NG}^{0}}$	Unstressed equilibrium constant	90.01	[25]
*y*_*I*_	Force compliance for two states ratio	8.16×10^−4^ nm	[25]

The bond dissociation kinetic parameters used in the MAD model are given here.

### Parameters evaluated and analysis metrics

Unless otherwise specified, the initial simulation condition was the cell oriented with its major axis parallel to the wall and the pseudopod oriented downstream in the direction of flow. A range of physiologically relevant shear rates, bond formation rates, receptor densities, and initial positions were tested on this cell geometry. The default bond formation rate used was 10 s^-1^, as this produced realistic behavior and was close to the value used in previous neutrophil MAD studies [15]. Model outputs at each time step included: translational and rotational position; node contact area and time; individual bond formation and breakage events; and bond forces and lifetimes. A collision event was assumed to have occurred if any part of the cell came within a reactive distance of the endothelial surface; here, it was set at 0.58 μm, the total length of a P-selectin/PSGL-1 bond plus the steric layers. The simulations were stopped when either the centroid of neutrophil reached 150 μm on the *x*-axis or a maximum number of bonds (200) were reached and the neutrophil remained firmly stuck to the endothelium. Contact time was calculated as the total amount of time any part of the cell was within potential binding range of the endothelium. Contact area was defined as the surface area of the cell that lied within a reactive distance to the endothelial surface, with or without binding events. The time integral contact area was the integral of the product of contact time and contact area.

### *In vivo* imaging of neutrophil-endothelial cell interactions

The simulation results were qualitatively compared to neutrophil adhesive behavior *in vivo* in intact, blood perfused cremaster muscle venules. Neutrophil-endothelial cell interactions in unstimulated venules of 30–60 μm diameter in anesthetized mice were visualized as described previously [31]. Briefly, neutrophils were visualized using an anti-Ly6G antibody conjugated to Alexa488 (1A8, Biolegend, 1.5μg/mouse) injected intravenously. Fluorescence images were digitally acquired at a frame rate of 10 fps, using an intensified CCD camera (XR Mega 10, Stanford Photonics, Palo Alto, CA) attached to a Nipkow spinning disk confocal head (CSU 10, Yokogawa Yokogawa Electric, Tokyo, Japan), and following tissue illumination with a 50mW argon laser. All mice were used according to protocols approved by the University of Rochester Institutional Animal Care and Use Committee.

## Results

### Selectin adhesion alters trajectory of neutrophil with pseudopod

**Fig 2:**
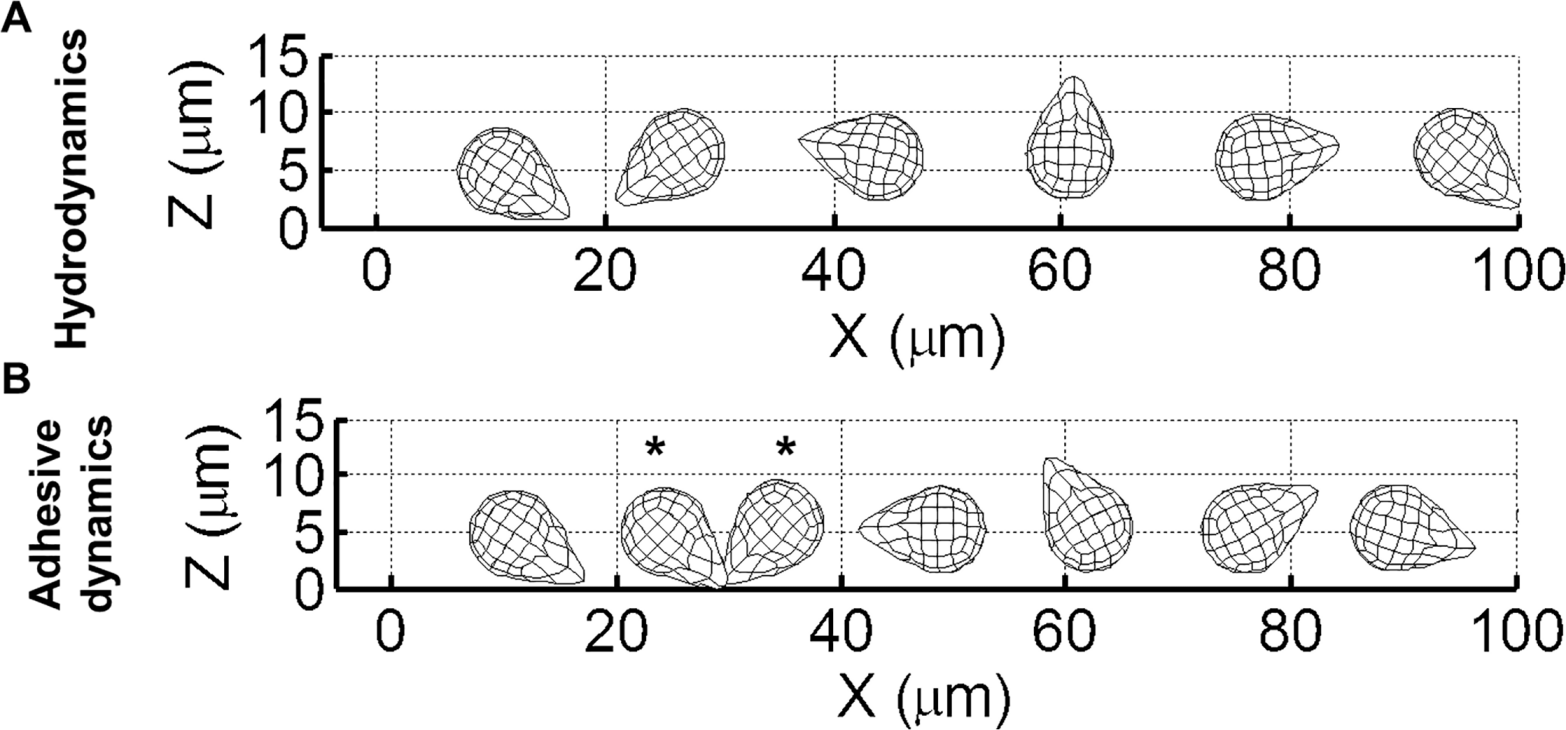
Comparison of side view trajectories of hydrodynamic and MAD simulations run under the same conditions. The shear rate is 1000 s^-1^ and the initial height is 0.5 μm. (A) and (B) are side views of the trajectories for hydrodynamic and P-selectin/PSGL-1 adhesion-enabled simulations, respectively. The asterisks in (B) indicate when bonds exist between the neutrophil and the endothelium, during which time the neutrophil is tethering.

**Fig 3:**
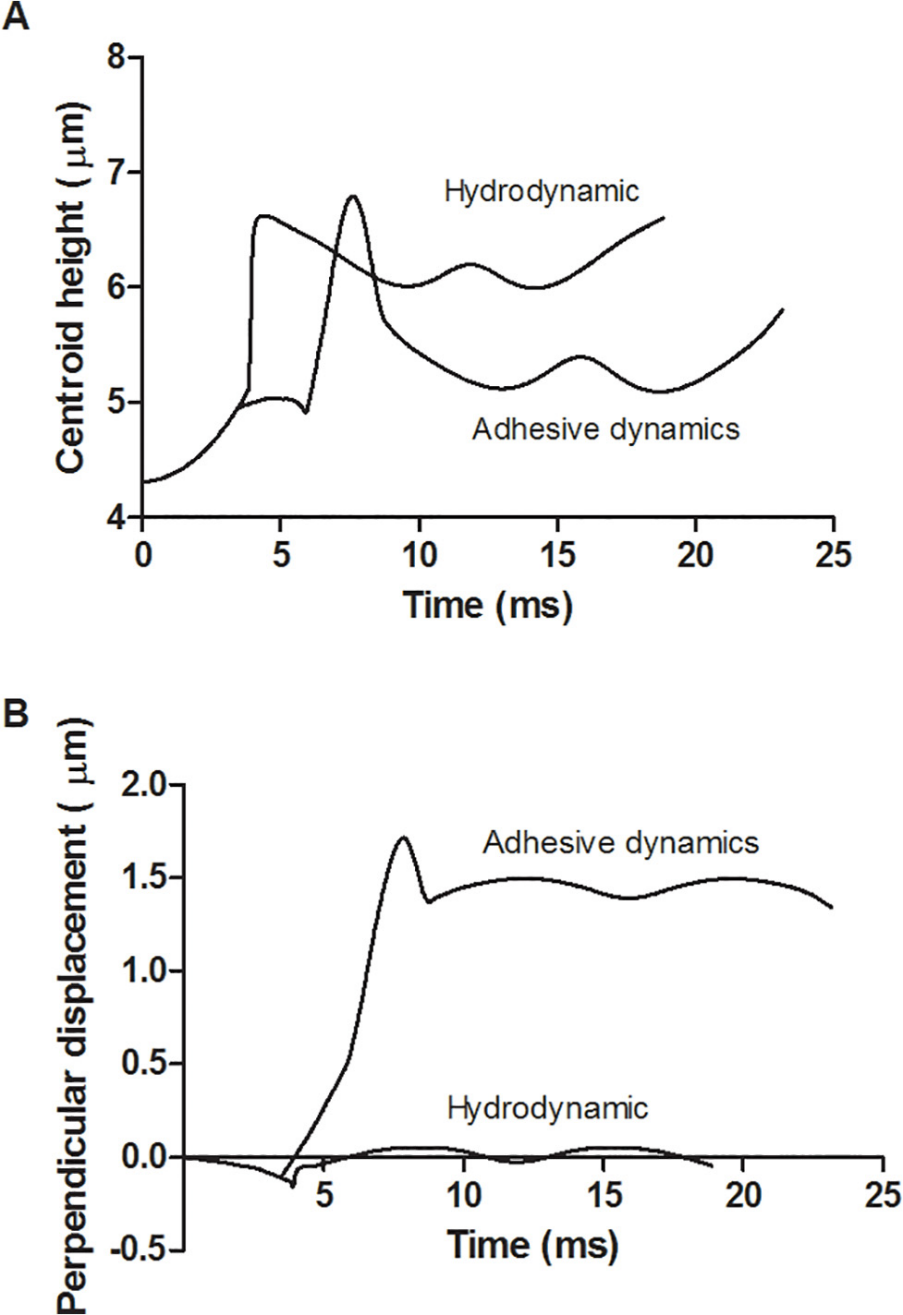
Comparison of hydrodynamic and adhesion-enabled simulations run under the same conditions. The shear rate is 1000 s^-1^, the dimensionless length is 1.9, and the initial height is 0.5 μm. The centroid heights for hydrodynamic and adhesion-enabled simulations are graphed versus time (A). The displacement perpendicular to the flow direction for both simulations is graphed versus time (B).

Representative trajectories for neutrophils with and without adhesion enabled (Fig 2A and Fig 2B, respectively) were compared. The simulations were carried out for a shear rate of 1000 s^-1^ and initial height of 0.5 μm. Without adhesion, the neutrophil pseudopod makes contact with the wall and the body then flips smoothly with very small deviation in the y-axis direction. With P-selectin/PSGL-1 adhesion, however, the neutrophil’s translation in the x-direction is slowed down as there is more pushing of the pseudopod prior to flipping (Fig 3A). When bonds form between the pseudopod and the endothelium, the neutrophil’s body swings around in the y-direction perpendicular to flow instead of parallel with the x-axis. Thus, the neutrophil’s overall centroid height is lower (Fig 3A) and its perpendicular displacement is greater Fig 3B) with adhesion.

### Characterizing neutrophil interaction behavior

The simulated neutrophil interaction behaviors were found to fall into one of several categories:

1. Firm adhesion upon first contact with the endothelium–A bound neutrophil that reached or approached the maximum number of bonds was assumed to be stationary, or “firmly adhered” to the endothelial wall. Physiologically, a neutrophil that was firmly adhered would likely begin crawling along and/or extravasating through the endothelium, but the model does not allow for the neutrophil to deform and exit the bloodstream. Typically, adhered neutrophils were found to be oriented with their body downstream and pseudopod upstream.
2. Tethering to the endothelium before subsequent firm adhesion–Tethering is defined here as behavior in which the neutrophil briefly forms bonds with the endothelium and detaches shortly thereafter.
3. Tethering without firm adhesion in the positional range studied.
4. No bonds formed.

Representative displacements over time for each behavior that formed bonds are shown in S1 Fig. All behavior types displayed very similar displacement trajectories until about *t* = 12 ms, when the adhesion cases form bonds with the endothelium via their pseudopods. Dragging of the pseudopod, as seen in Fig 2B, leads to decreased velocity. In contrast to the tethering simulations, the firm adhesion cases exhibited at least one bond with the endothelium until around *t* = 20 ms, when the neutrophil body had flipped over the pseudopod and remained stuck at around *x* = 35 μm for the remainder of the simulation. In the tethering events, the neutrophil body flipped over the bound pseudopod and detached at around *t* = 20 ms, displaying constant velocity afterward. In the cases where one or two tethering events occurred before adhesion, the velocity was reduced at about *t* = 50 ms, where the neutrophils remained stuck to the endothelium at a position of around *x* = 105 μm. The case in which no bonds form did not exhibit reduced velocity at *t* = 12 ms and remained at a relatively constant speed throughout the simulation.

### Tethering is conserved across a range of physiological shear rates

**Fig 4:**
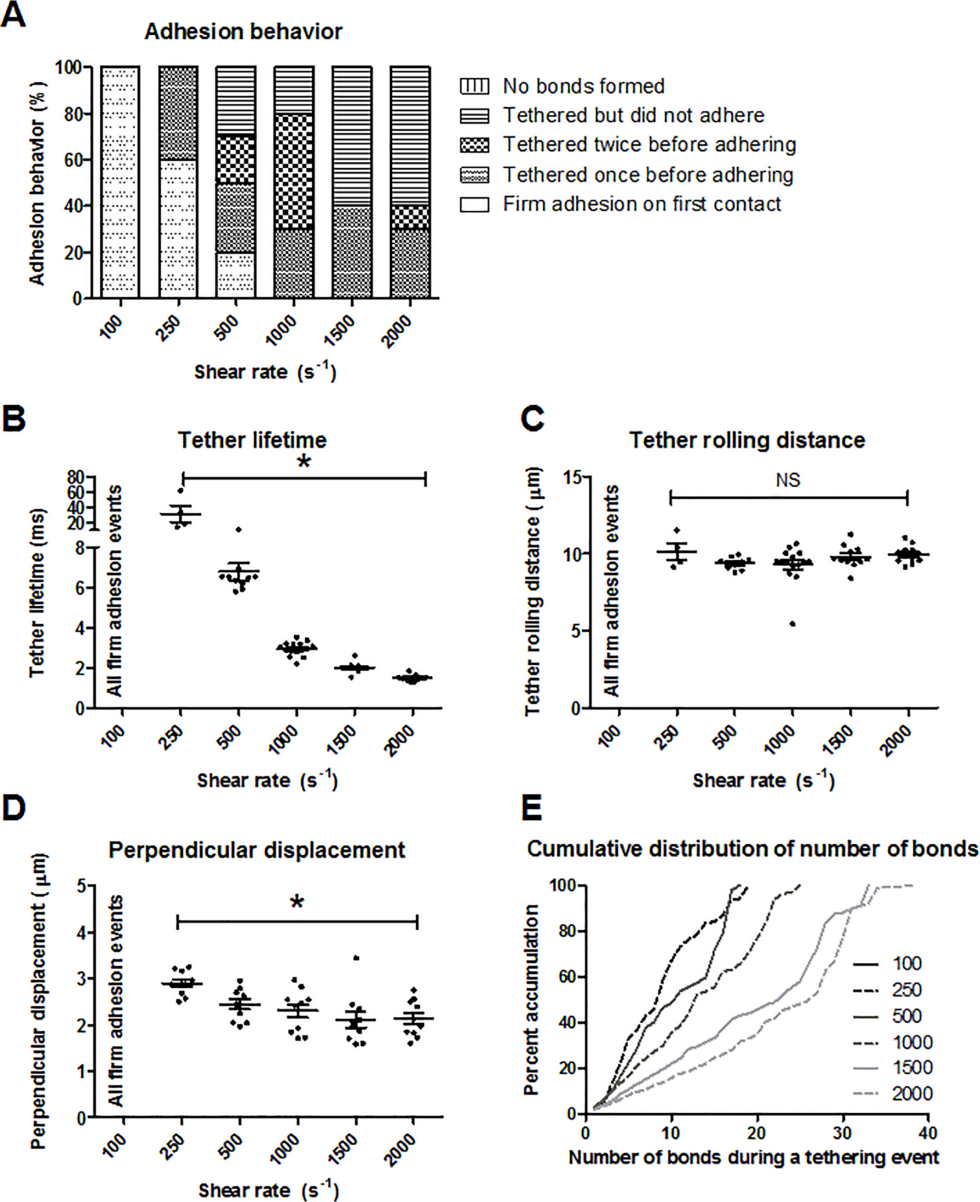
Effect of shear rate on adhesion interactions. (A) Characterization of adhesion behavior, (B) tether lifetime, (C) tether rolling distance, (D) centroid displacement perpendicular to flow, and (E) cumulative frequency distribution for varying shear rates. Ten runs were performed for each condition. *p<0.01, and the mean and standard error are shown.

When P-selectin/PSGL-1 adhesion is included in MAD, the shear rate affects the adhesive behavior. Neutrophil simulations were run at increasing shear rates of 100 s^-1^, 250 s^-1^, 500 s^-1^, 1000 s^-1^, 1500 s^-1^, and 2000 s^-1^ starting at an initial height of 0.5 μm. The bond formation rate used was 10 s^-1^, and ten runs were performed at each shear rate. At a shear rate of 100 s^-1^, all of the neutrophils firmly adhered to the endothelium upon first contact (Fig 4A). At higher shear rates, more tethering occurred prior to firm adhesion as well as an increase in tethering without adhesion. There were bonds formed during all of the simulations for each shear rate studied. Tethering lifetimes and rolling distances were examined (Fig 4B and 4C, respectively). Tether lifetime was defined as the total time from the neutrophil’s first bond with the endothelium until detachment of all bonds from the endothelium. Tether rolling distance was the total distance in the flow direction during which the neutrophil held at least one bond with the endothelial wall. There was no tethering behavior at a shear rate of 100 s^-1^ as 100% of the runs resulted in firm adhesion upon first contact with the endothelium. Tether lifetime decreased nonlinearly at higher shear rates (Fig 4B). An 8-fold increase in shear rate from 250 s^-1^ to 2000 s^-1^ resulted in a 29.9% decrease in mean tether lifetime. An increase in shear rate from 500 s^-1^ to 2000 s^-1^ produced little change in mean tether rolling distance (Fig 4C). At higher shear rates, less perpendicular displacement was observed (Fig 4D). While the pseudopod was bound to the endothelium, the neutrophil’s body swung around in the y-direction perpendicular to flow instead of parallel with the x-axis. A 20-fold increase in shear rate from 100 s^-1^ to 2000 s^-1^ resulted in a 39.4% decrease in the mean maximum centroid displacement in the y-direction. As tether lifetime decreases, perpendicular displacement of the neutrophil body also decreases; the neutrophil is making less contact with the endothelium. The cumulative frequency distribution for the number of bonds at each shear rate is shown in Fig 4E. This compiles the number of bonds at each time point during all tethering events of a simulation. The cumulative frequency curves were relatively linear over the range of shear rates studied. Also, the maximum bond number increased linearly (*R* ^2^ = 0.920) by 100% from a shear rate of 250 s^-1^ to 2000 s^-1^.

### Firm adhesion requires higher bond formation rate

**Fig 5:**
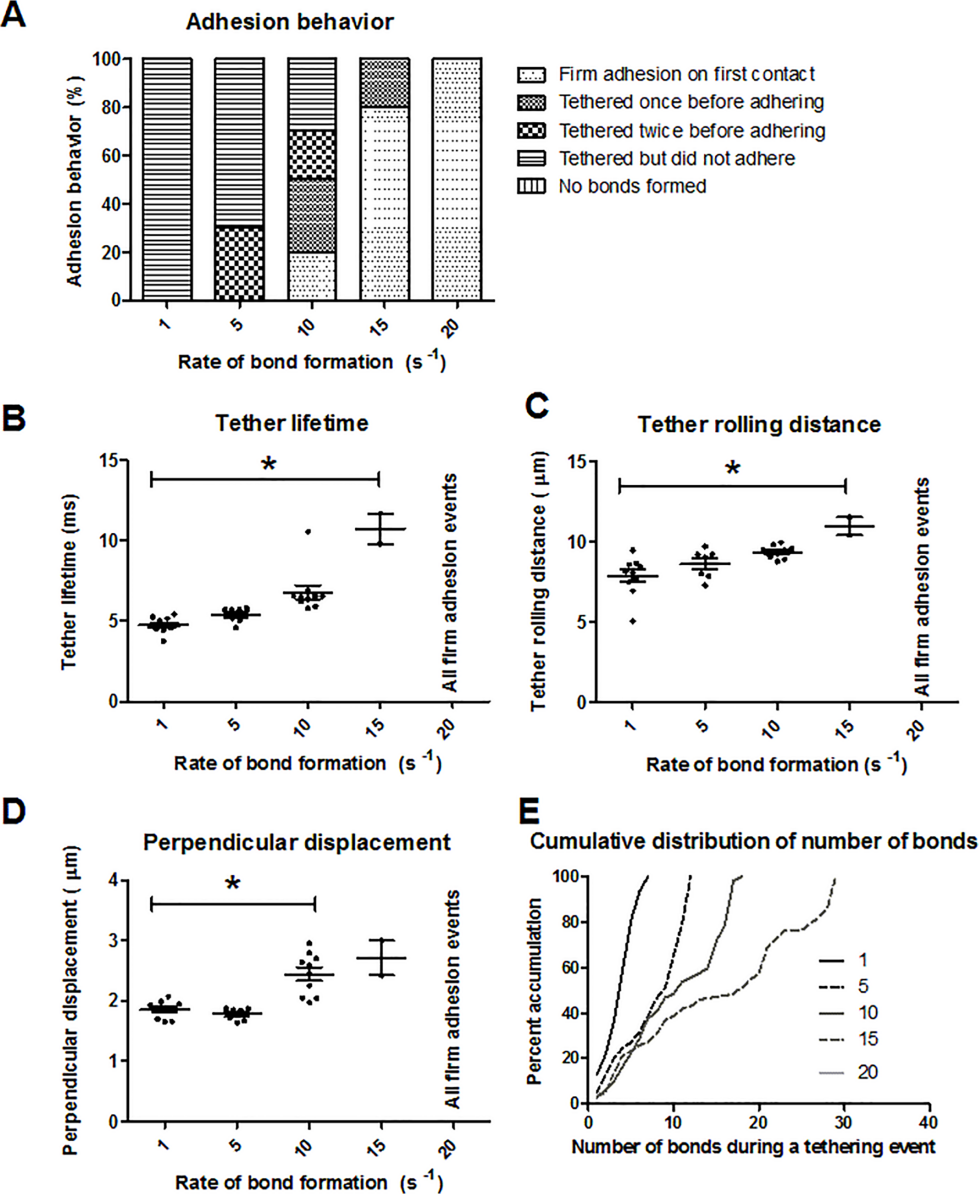
Effect of bond formation rate on adhesive interactions. (A) Characterization of adhesion behavior, (B) tether lifetime, (C) tether rolling distance, (D) centroid displacement perpendicular to flow, and (E) cumulative frequency distribution for varying bond formation rates. Ten runs were performed for each condition. *p<0.01, and the mean and standard error are shown.

The effect of the rate of P-selectin/PSGL-1 bond formation on rolling and tethering behavior was studied. A neutrophil of length 1.9 was simulated at increasing bond formation rates of 1, 5, 10, 15, and 20 s^-1^ starting at an initial height of 0.5 μm. The shear rate used was 500 s^-1^. All bond formation rates studied resulted in bond formation for all simulations. At a bond formation rate of 1 s^-1^, 100% of the simulations resulted in tethering events with no adhesion (Fig 5A). As bond formation rate increased, there was an increase in the number of firm adhesion events. At bond formation rates of 10 s^-1^, 15 s^-1^, and 20 s^-1^, firm adhesion occurred upon first contact for 20, 80, and 100% of all simulation cases, respectively. Mean tether lifetime and tether rolling distance increased from 1 to 15 s^-1^ by 126.2% and 39.3%, respectively (Fig 5B and 5C). Similarly, the displacement of the neutrophil’s centroid perpendicular to flow increased from 1 to 10 s^-1^ by 32.0% (Fig 5D). The cumulative frequency curves of the number of bonds at each time point during tethering were mostly linear for all bond formation rates studied (Fig 5E). The maximum number of bonds at one time during a tethering event increased exponentially (*R* ^2^ = 0.997) as bond formation rate was increased, showing a total increase of 314.2% from *k*_*f*_^*0*^ = 1 to 15 s^-1^.

### Lower receptor counts correspond to decreased tether lifetime

**Fig 6:**
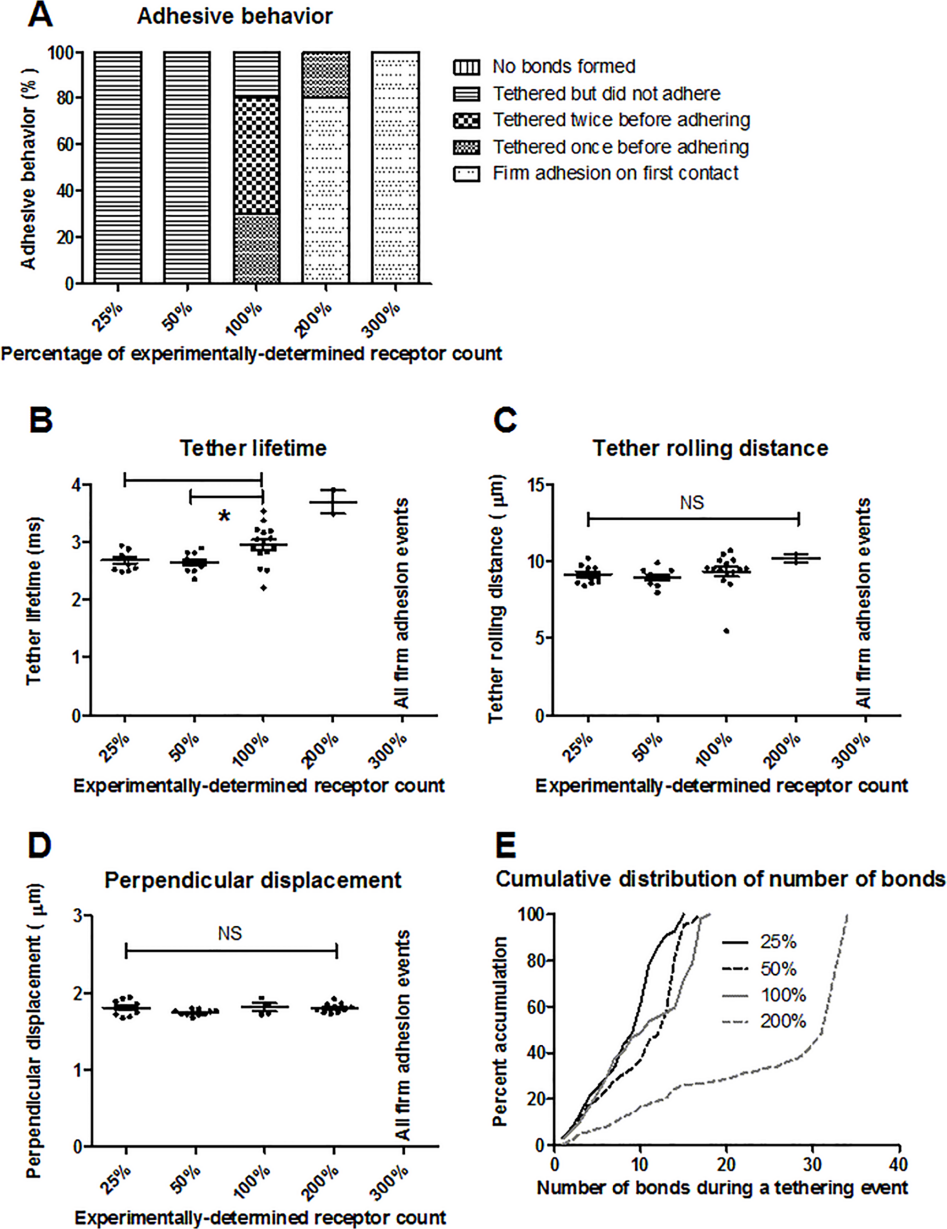
Effect of receptor number on adhesive interactions. (A) Characterization of adhesion behavior, (B) tether lifetime, (C) tether rolling distance, (D) centroid displacement perpendicular to flow, and (E) cumulative frequency distribution for varying receptor numbers relative to the experimentally-determined value. Ten runs were performed for each condition. *p<0.01, and the mean and standard error are shown.

In addition to shear rate, sensitivity analysis was performed by varying the number of PSGL-1 receptors (25, 50, 200, and 300%) on the neutrophil’s surface. As with the experimentally-determined receptor number, the increased and decreased receptor counts were distributed homogeneously across the neutrophil surface. The P-selectin/PSGL-1 bond formation rate was 10 s^-1^, the shear rate used was 500 s^-1^, and the neutrophil’s initial height was 0.5 μm. The rolling behavior at each on-rate was examined (Fig 6A). For 25 and 50% receptor counts, all of the simulation runs showed tethering but no firm adhesion. For the 200 and 300% receptor counts, there were increasing instances of firm adhesion, whereas there was no firm adhesion observed for the experimentally-determined number of receptors. As shown in Fig 6B, small decreases were observed in the tether lifetime for the 50 and 25% receptor counts (11.4 and 9.9%, respectively) compared to the physiological receptor count. There was no significant difference among tether rolling distances when the receptor number was varied (Fig 6C). Regarding the displacement of the neutrophil’s centroid perpendicular to flow, there was no significant difference between the base case compared to the other receptor densities (Fig 6D). For 50 and 25% receptor counts, the cumulative distributions of the number of bonds during tethering events were similar to the base case (Fig 6E). The maximum number of bonds formed during tethering events for the 200% receptor case was almost twice as high (increase of 88.9%) as for the base case (34 vs. 18 bonds, respectively). No tethering was observed for the 300% receptor case since all simulations resulted in firm adhesion upon first contact.

### Most adhesion occurs on the side of pseudopod

**Fig 7:**
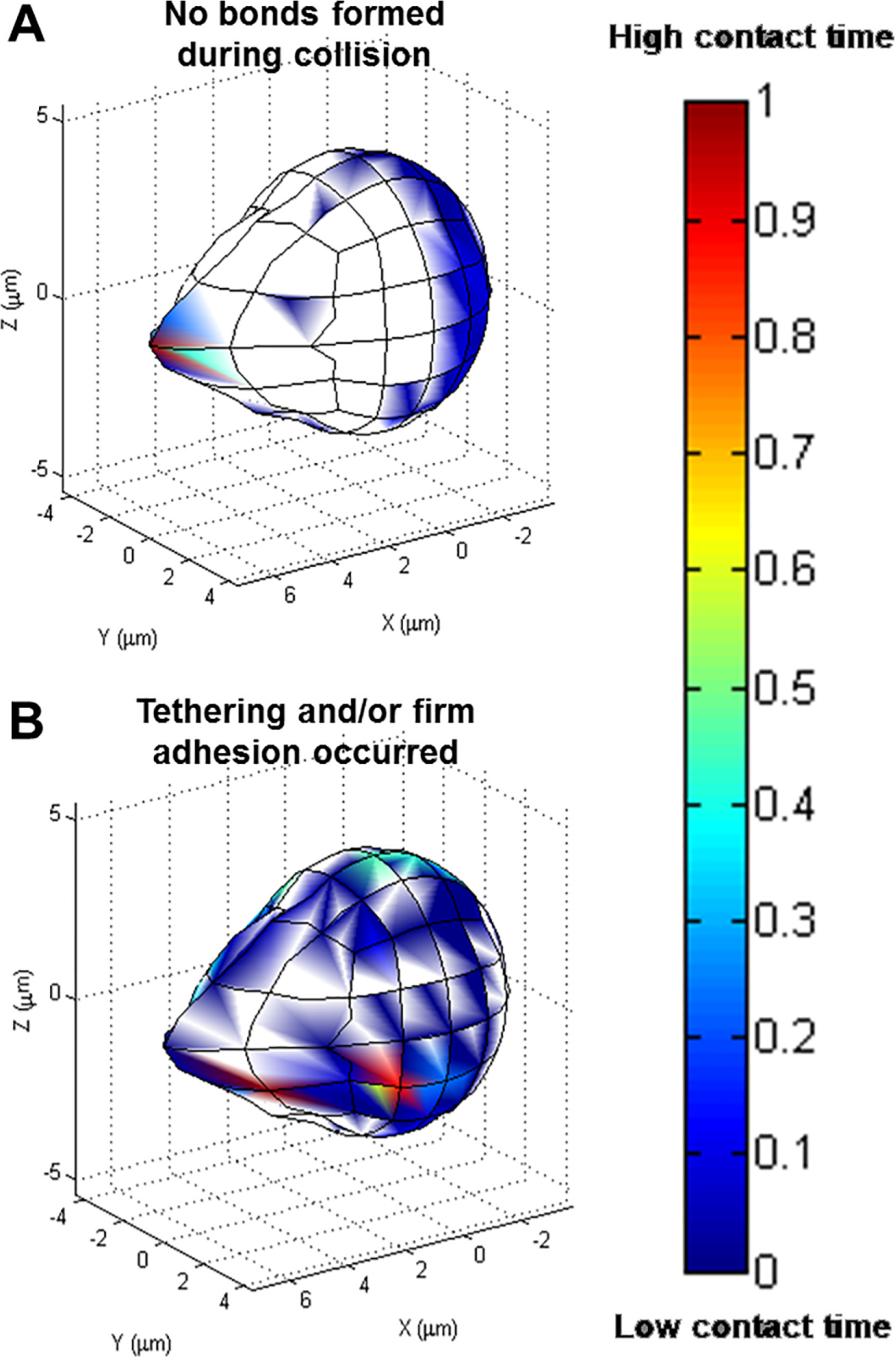
Relative contact time for collisions where bonds did and did not occur. Relative contact time for collisions where (A) no bonds formed and (B) adhesion events occurred, pooled from the collisions of >60 initial angles. Red indicates the longest contact time, and blue is the shortest contact time.

**Fig 8:**
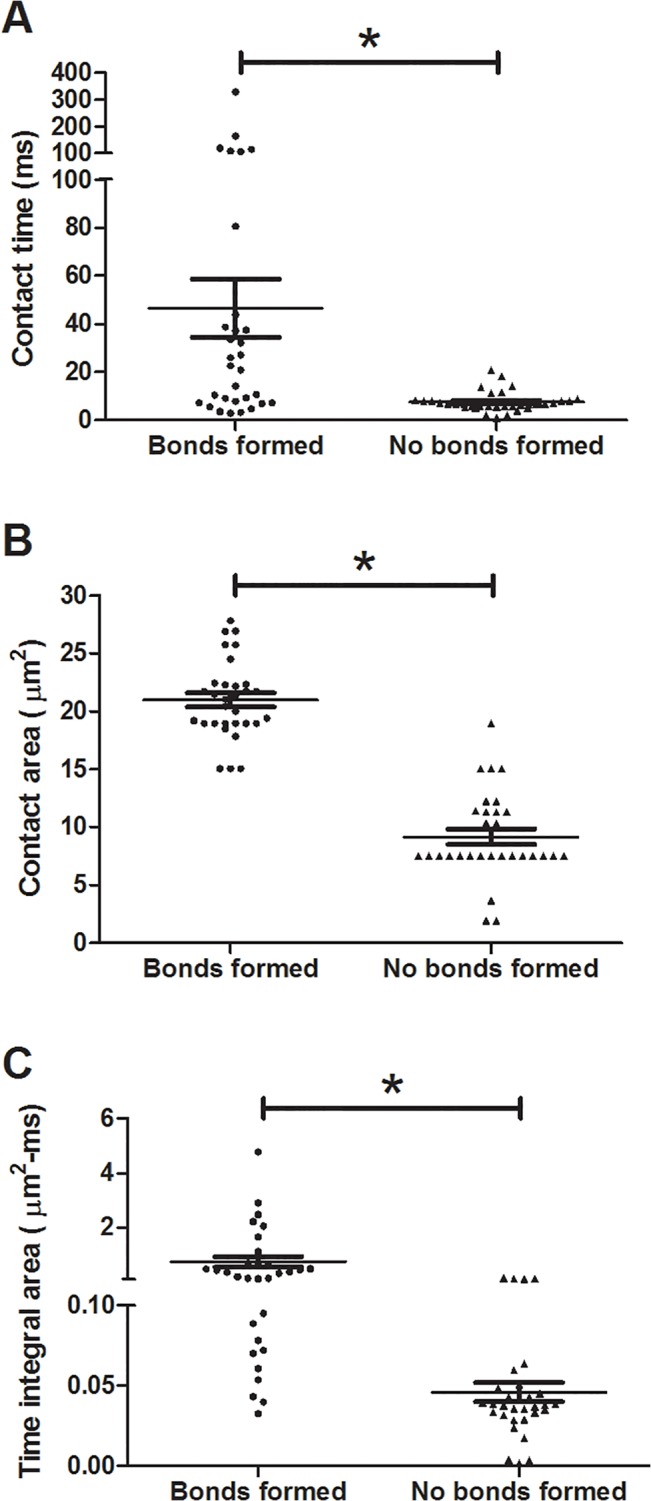
Contact time and contact area for collisions where bonds did and did not occur. Contact time (A), contact area (B), and time integral contact area (C) graphs for collisions where no bonds formed and where adhesion events occurred. *p<0.01.

To determine where adhesion was most likely to occur on the neutrophil’s surface, a range of initial angles was studied. The angle combination ranges were 0 to π at π/4 intervals (for a total of 125 angles), and angles resulting in wall penetration were excluded. For this set of simulations, the neutrophil’s centroid height was 1 μm off the endothelial surface, the shear rate was 500 s^-1^, and the bond formation rate used was 10 s^-1^. The initial height was chosen as a compromise between more frequent collision occurrence and with relatively fewer neutrophil positions excluded due to steric overlap with the wall. When no bonds formed, contact on the neutrophil’s surface was focused at the pseudopod tip (Fig 7A). In contrast, when the neutrophil tethered or firmly adhered to the endothelial surface, the area of contact was located along the side of the pseudopod (Fig 7B). The metrics examined for these behaviors were the contact time, contact area, and time integral contact area (Fig 8A, 8B and 8C, respectively). Adhesion and/or tethering events showed significantly higher mean contact time and contact area compared with simulation runs where no bonds formed between the neutrophil and the wall, 46.62 ms and 20.99 μm^2^ vs 7.530 ms and 9.181 μm^2^. Likewise, time integral contact area showed a similar trend, 0.75 μm^2^-ms for runs with bonds forming and 0.046 μm^2^-ms for runs without bonds. Contact time contributed greater variation to time integral contact area than did contact area.

### Behavior of tethering neutrophils with pseudopods in vivo resembles simulation results

**Fig 9:**
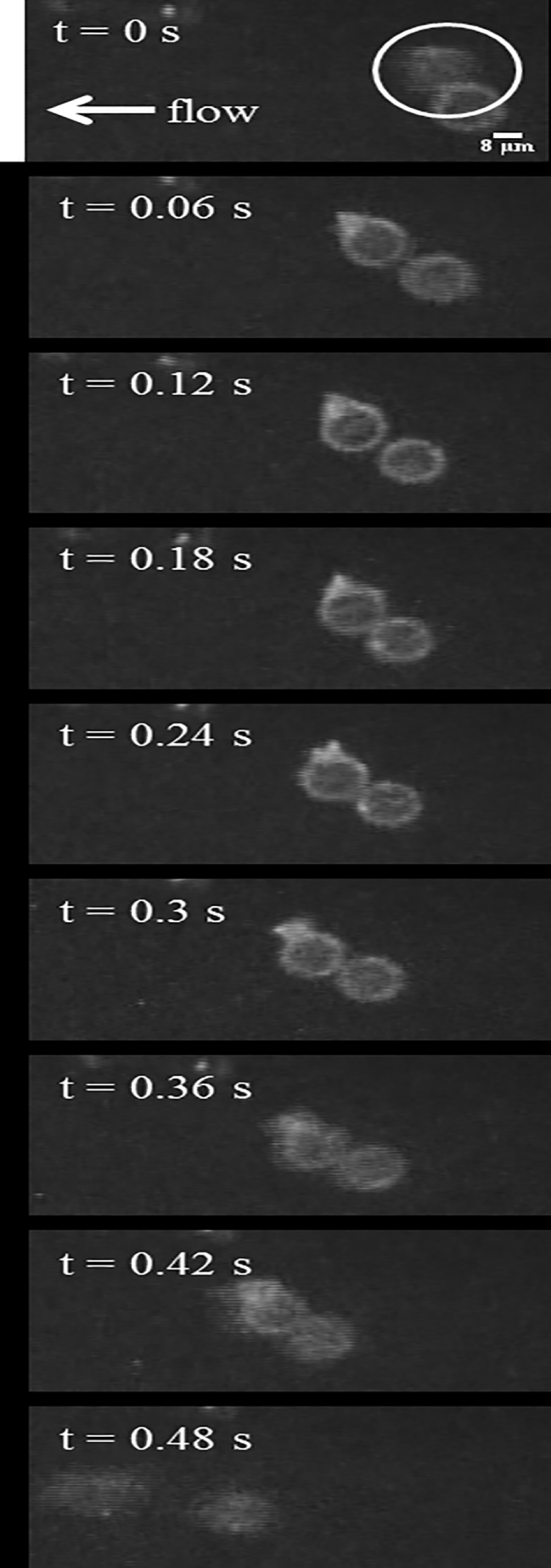
*In vivo* example of neutrophil tethering in a blood perfused cremaster muscle venule. The circled neutrophil is observed to tether to the endothelium from *t* = 0.06 s until *t* = 0.42 s, after which it detaches and rejoins the blood flow. The images were acquired using fluorescent confocal microscopy.

**Fig 10:**
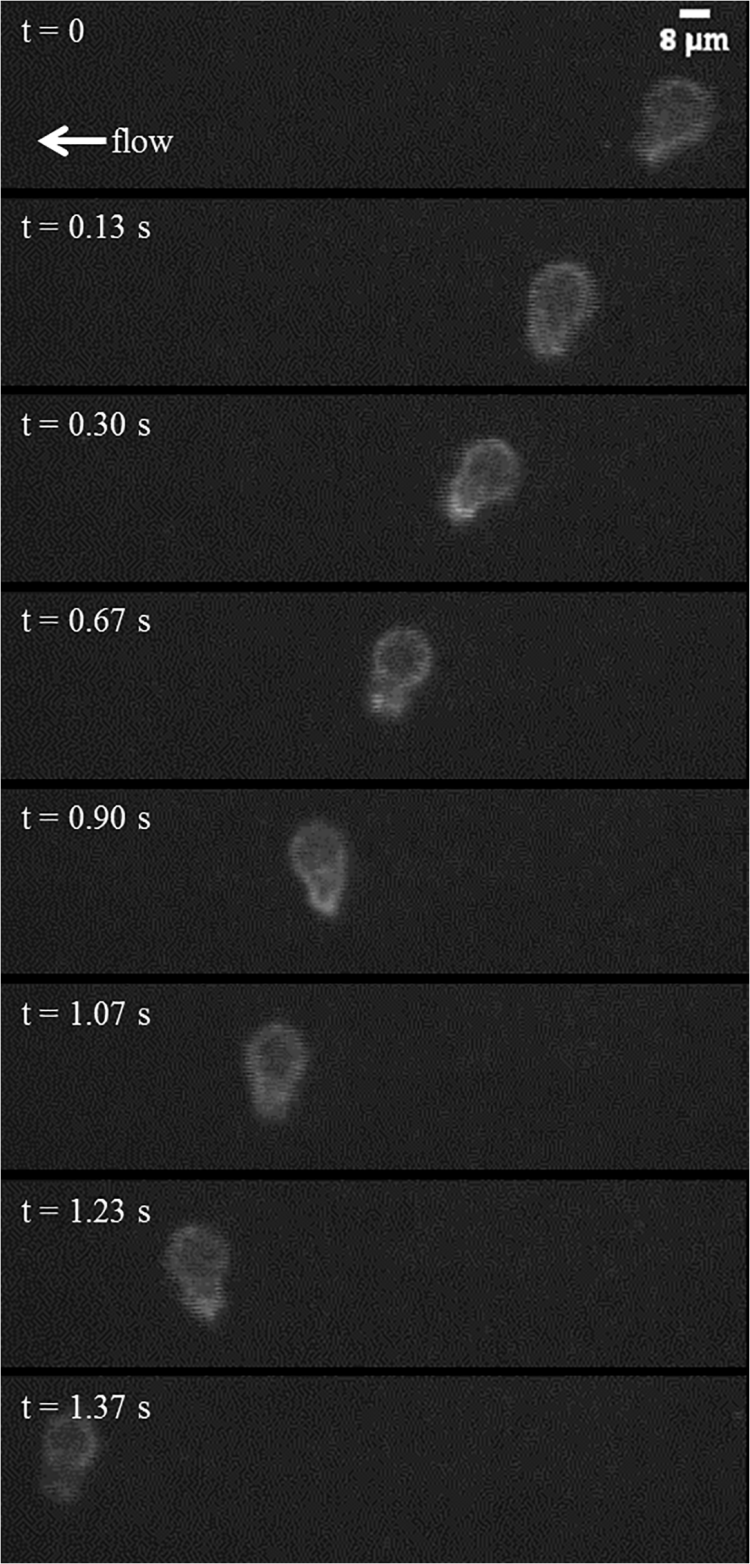
Time-lapse images of a rolling neutrophil in a blood perfused postcapillary venule. A representative neutrophil tethers to the endothelium at *t* = 0.13 s and detaches by *t* = 1.37 s to rejoin the blood flow.

**Fig 11:**
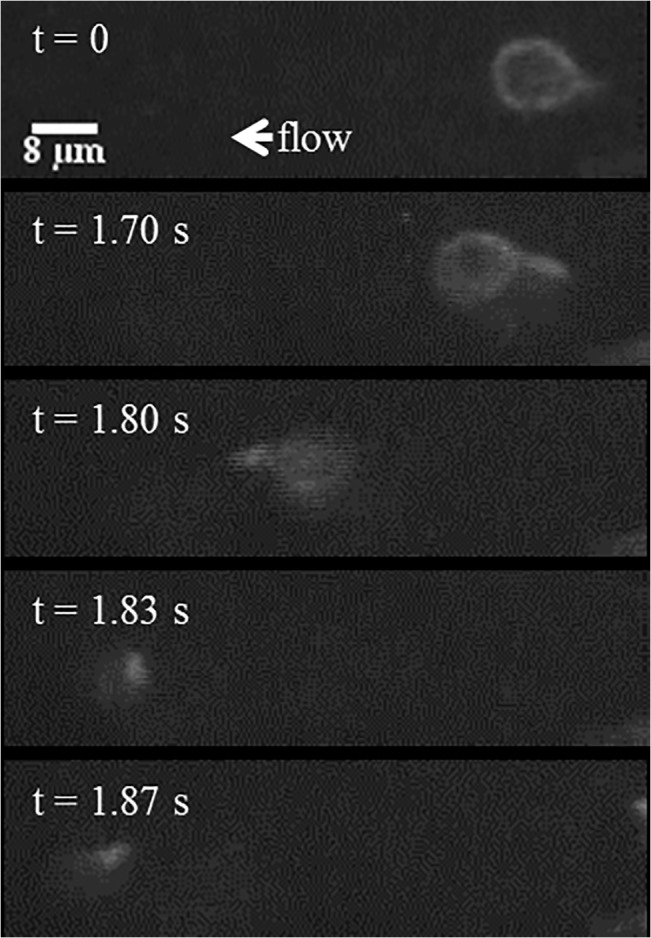
*In vivo* sequence depicting a representative, Ly6G labeled neutrophil with a long pseudopod. It detaches, moves downstream with the pseudopod flipping around, and reattaches via the existing pseudopod.

**Fig 12:**
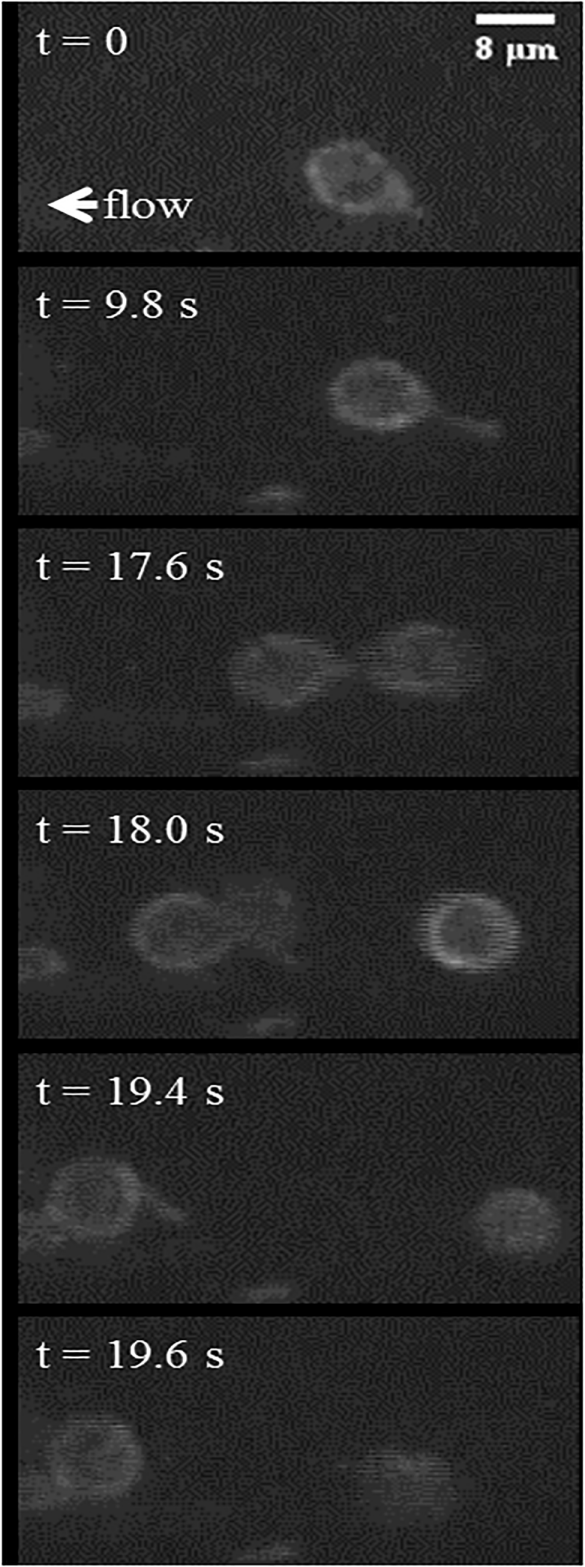
Time-lapse images show a representative, activated neutrophil as it slowly rolls along the vascular wall. The rolling neutrophil (*t* = 0 s) is observed to slow down through an extension of a pseudopod attached to the vascular endothelium (*t* = 9.8 s). It continues rolling by disconnecting the pseudopod to rejoin the flow (*t* = 17.6 s), reattaches to the endothelium using the same pseudopod (*t* = 18 s), and finally disconnects the pseudopod (*t* = 19.4), rapidly retracting it into the cell body and continues to roll along the endothelium (*t* = 19.6 s).

The tethering behavior exhibited in the MAD model is qualitatively consistent with *in vivo* data of neutrophils with pseudopods in the bloodstream in healthy mice. In the representative events shown in Fig 9 and Fig 10, a neutrophil with stable pseudopod attaches to the endothelium and swings its body around until it detaches and rejoins the blood flow. Fig 11 shows a Ly6G labeled neutrophil with a long pseudopod as it detaches, moves downstream with the pseudopod flipping around, and reattaches via the existing pseudopod. Fig 12 shows a neutrophil as it extends a long pseudopod, disconnects the pseudopod and joins the flow, reattaches downstream via the existing pseudopod, disconnects the pseudopod, and rapidly retracts the pseudopod. A representative video is given in S1 Video. Overall, the trajectory resembles the simulation trajectories obtained through simulation. In both the *in vivo* and simulation examples, the pseudopod is the primary area of contact on the neutrophil that enables adhesion to the endothelium, and the attachment is transient.

## Discussion

Several types of behavior were observed in the simulations, comprising: tethering without firm adhesion, tethering with downstream firm adhesion, and firm adhesion upon first contact with the endothelium. The tether lifetime observed in the simulations for lower shear rates was on the order of that found for a white blood cell with single microvillus at a shear stress of 1 dyn/cm^2^, ~30 ms [32]. During simulation adhesion events, the preferred orientation of the neutrophil (pseudopod upstream, body downstream) resembled the geometry and orientation of a deformable adhered leukocyte [33–38]. This deformed, tear drop geometry produces less drag on adherent leukocytes than rigid spheres. The *in vivo* images show several examples of neutrophils with stable pseudopods tethering once or twice without firm adhesion as well as an example of a neutrophil with a pseudopod that tethers before retracting its pseudopod. There is great variation among pseudopod shapes [7], and it would be interesting to study more geometries. For instance, does a longer pseudopod have greater contact time during an adhesion event than a shorter, wider pseudopod of equal volume? Furthermore, there are often multiple pseudopods on a single cell [7,39], and an examination of their relationship to one another would encompass a greater number of realistic scenarios.

In this model, the PSGL-1 receptors are uniformly distributed across the neutrophil’s surface. Biologically, receptors can be clustered on the tips of microvilli, making the effective concentration of receptors locally higher. Furthermore, receptors can be found to be more dense on the pseudopod than on the neutrophil body [40], for which our study does not account. Chang and Hammer discussed how receptor sensitivity accounts for heterogeneity in receptor counts among neutrophils [18]. A MAD model developed for deformable microvilli with receptor clustering utilized a comparable receptor density (4 microvilli per μm^2^, 24 receptors per microvillus) for an equivalent spherical cell volume [41]. The current results showing that the neutrophil’s tethering behavior resists changes in receptor count corroborate this finding. However, due to the heterogeneity among neutrophils, and the fact that most binding occurs on the pseudopod, it is likely that the range of behaviors may be captured by increasing and decreasing the overall receptor density.

Increasing the shear rate was found to increase the incidence of firm adhesion upon first contact; a shear threshold phenomenon was not observed here. However, catch-slip kinetics do not necessarily lead to larger-scale cell rolling that exhibits the shear threshold phenomenon [42]. It is expected that there would be changes to the rolling and adhesion behavior if other selectin binding kinetics were used in place of, or as a complement to, P-selectin/PSGL-1. For instance, E-selectin/PSGL-1 binding typically occurs later than P-selectin/PSGL-1 during inflammation [43]. L-selectin/PSGL-1 demonstrates a stronger shear threshold effect than P-selectin/PSGL-1 [42]. Tether rolling distance was found to be nearly constant for shear rates from 250–2000 s^-1^, which is similar to findings from Alon et al. [44].

## Conclusions

We have developed a three-dimensional computational model of a neutrophil with long pseudopod interacting with the endothelium under flow via PSGL-1/P-selectin catch-bond kinetics. In contrast to the transient endothelial contact exhibited in the hydrodynamic model, the neutrophil’s translation in the *x*-direction is slowed down due to P-selectin/PSGL-1 binding in the MAD model, leading to neutrophil tethering. While the pseudopod was bound to the endothelium, the neutrophil’s body swung around in the direction perpendicular to flow. Thus, the neutrophil’s overall centroid height is lower and its perpendicular displacement is greater with selectin binding than without selectin binding. This tethering behavior was found to be qualitatively consistent with *in vivo* data of murine neutrophils with pseudopods in the bloodstream. Tethering was conserved across a range of physiological shear rates and was resistant to changes in receptor count. Firm adhesion upon first contact with the endothelium increased as shear rate, receptor count, and bond formation rate increased. A range of initial orientations were tested, and simulations in which tethering events occurred experienced the greatest contact along the side of the neutrophil pseudopod. In contrast, in simulations where endothelial collisions occurred but no bonds formed, contact was focused on the pseudopod tip. The side of the pseudopod offered greater surface area to bind to the endothelial wall as well as greater contact time in the simulations where adhesion occurred was observed. This paper presented the effect of P-selectin and PSGL-1 binding on the hydrodynamics of neutrophil collisions with the endothelium. Future work should focus on pseudopod deformability and multiple pseudopods.

## Supporting Information

S1 Fig Representative displacements in the flow direction over time for tethering and adhesion behaviors. The neutrophil pseudopod length was 1.9, the initial height was 0.5 μm, the bond formation rate was 10 s^-1^, and the shear rate was 500 s^-1^.
(TIF)

S1 Video Time-lapse video showing examples of neutrophil adhesion to a mouse venule via pseudopods. The recording was done at a frame rate of seven frames per second.
(MOV)
