# Supplementary figures and images for: Simulation and Analysis of Tethering Behavior of Neutrophils with Pseudopods
Source: PLoS One. 2015 Jun 19;10(6):e0128378. doi: 10.1371/journal.pone.0128378 (PMC4474963; doi:10.1371/journal.pone.0128378)

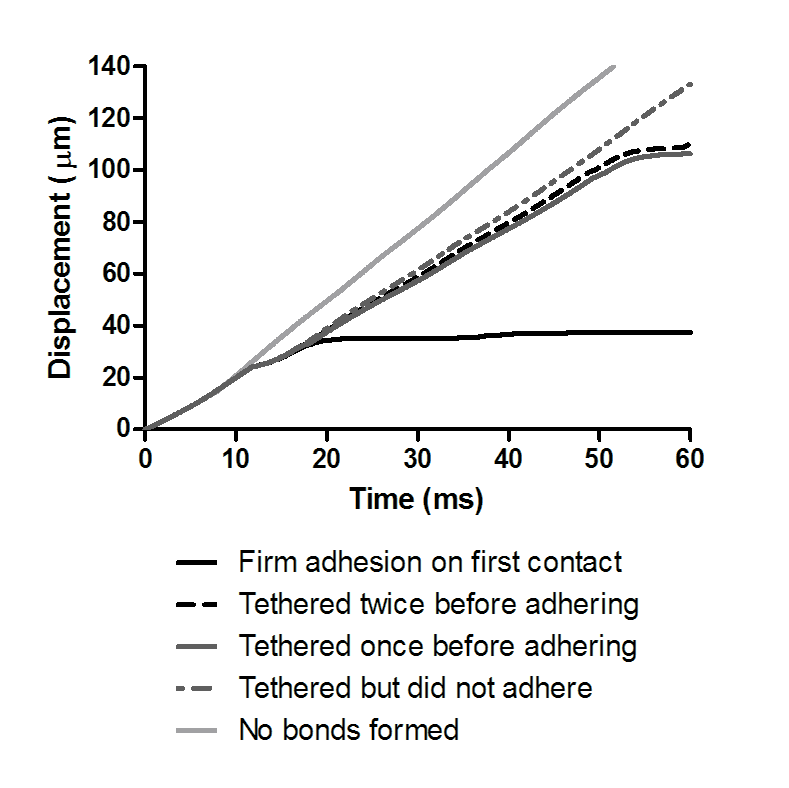

Supplement: S1 Fig — The neutrophil pseudopod length was 1.9, the initial height was 0.5 μm, the bond formation rate was 10 s-1, and the shear rate was 500 s-1. (TIF) [file pone.0128378.s001.tif]
